# Ureteral stent symptoms: A systematic review and meta‐analysis comparing the use of mirabegron and tamsulosin

**DOI:** 10.1002/bco2.485

**Published:** 2025-09-11

**Authors:** Daniel Madarshahian, Abdulrasheed Habeeb, Nimeshan Chandra‐Segaran, Kesavapilla Subramonian, Keval Patel

**Affiliations:** ^1^ Department of Urology University Hospitals Birmingham NHS Foundation Trust Birmingham UK; ^2^ Department of General Surgery University Hospitals Birmingham NHS Foundation Trust Birmingham UK

**Keywords:** meta‐analysis, mirabegron, tamsulosin, ureteral stent‐related symptoms

## Abstract

**Background:**

Ureteral stent insertion, crucial for managing ureteral obstructions, often results in stent‐related symptoms (SRSs) adversely affecting patient quality of life. This meta‐analysis compares the effectiveness of tamsulosin or mirabegron versus placebo in alleviating these symptoms.

**Methods:**

Following PRISMA guidelines, we systematically reviewed randomized controlled trials (RCTs) comparing mirabegron or tamsulosin to placebo in managing SRSs. Data sources included PubMed, Embase, Web of Science and CENTRAL, up to November 2023. The inclusion criteria focused on studies reporting on Ureteral Stent Symptom Questionnaire (USSQ), International Prostate Symptom Score (IPSS), quality of life (QoL) assessments, analgesic usage and adverse events. Meta‐analysis employed a random‐effects model, assessing heterogeneity and publication bias. For assessing the risk of bias in the included randomized trials, we employed the Cochrane Collaboration's tool. This protocol was registered at the International Prospective Register of Systematic Reviews (registration number: CRD42024511842).

**Results:**

Sixteen RCTs with 1635 patients met the inclusion criteria. Tamsulosin significantly improved body pain (MD −1.80; 95% CI −3.53 to −0.07; *p* = 0.04), sexual function (MD −0.63; 95% CI −1.16 to −0.10; *p* = 0.02) and improved quality of life score (MD −2.36; 95% CI −3.56 to −1.17; *p* = 0.0001), while mirabegron was more effective in reducing urinary symptoms (MD −8.71; 95% CI −15.81 to −1.61; *p* = 0.02), enhancing general health (MD −2.58; 95% CI −3.78 to −1.37; *p* < 0.0001) and reducing analgesia use (MD −1.56; 95% CI −2.70 to −0.41; *p* = 0.008). Both medications significantly reduced total International Prostate Symptom Score (Tamsulosin MD −8.4; 95% CI −15.63 to −1.22; *p* = 0.02; Mirabegron MD −6.29; 95% CI −8.50 to −4.08; *p* < 0.00001) without a significant rise in adverse events (tamsulosin OR 1.90; 95% CI 0.40–9.18; mirabegron *p* = 0.42 and OR 0.93; 95% CI 0.30–2.88; *p* = 0.89).

**Conclusions:**

Tamsulosin and mirabegron effectively manage SRSs, with distinct benefits in different symptom domains. This suggests a potential for complementary therapeutic strategies. Future high‐quality RCTs are needed to explore their combined efficacy.

## 1 INTRODUCTION

Ureteral double‐J stent insertion is a cornerstone in contemporary urologic therapeutics. While these stents are integral for managing ureter obstruction and aiding in pelvic surgeries, their widespread adoption is accompanied with significant morbidity. Patients commonly experience a spectrum of ureteral stent‐related symptoms (SRSs), including voiding and storage symptoms, flank pain, haematuria and infection, which detrimentally impact quality of life (QOL) in up to 80% of cases.^1^

The management of SRSs continues to pose a significant clinical challenge in urology. Tamsulosin, a selective alpha‐1a adrenergic receptor inhibitor, has been shown to effectively reduce urinary symptoms and pain and improve sexual performance compared to placebo.^2^, ^3^ Concurrently, mirabegron, a β3 adrenergic receptor agonist, has emerged as a promising alternative, demonstrating significant efficacy in treating ureteral SRSs and outperforming both placebo and blank controls.^4^ Although tamsulosin's efficacy in alleviating SRSs is well‐documented, its comparative effectiveness against mirabegron has not been extensively explored, highlighting the need for further investigation. This meta‐analysis aims to evaluate single randomized controlled trials (RCTs) to evaluate their effectiveness in managing SRSs. Specifically, it will focus on comparing the benefits of tamsulosin versus mirabegron against placebo across similar domains.

## 2 METHODS

### 2.1 Design and eligibility criteria

Selection of studies, data collection, outcome synthesis and data analysis were done according to prespecified criteria, which had been documented in a review protocol. This protocol was registered at the International Prospective Register of Systematic Reviews (registration number: CRD42024511842). The review conformed to the Preferred Reporting Items for Systematic Reviews and Meta‐Analyses (PRISMA) statement standards.^5^

We established specific criteria for the inclusion of RCTs in this meta‐analysis. The criteria are as follows: (a) The study must involve treatment with Tamsulosin or Mirabegron compared to placebo or blank controls for managing ureteral SRSs. (b) The study population should include participants of any age or gender who have undergone stone treatment, ureteroscopic procedures or ureteral surgery, including pyeloplasty. (c) Essential for inclusion is the availability of analysable data, which includes the total number of participants in each group and outcome values such as scores from the Ureteral Stent Symptom Questionnaire ([USSQ] consisting of urinary symptoms, body pain, general health, work performance and sexual matters), the International Prostate Symptom Score (IPSS), QOL assessments, analgesic usage and adverse events. (d) A minimum stent indwelling and follow‐up period of 1 week is required. (e) Studies must be available in full text and published in English. Studies not adhering to these eligibility criteria were excluded from the analysis.

### 2.2 Search methods

A comprehensive search was conducted for publications up to 19 September 2024, encompassing major databases including PubMed/MEDLINE, Google scholar, Embase, Web of Science and the Cochrane Central Register of Controlled Trials (CENTRAL). This search was supplemented by reviewing reference lists from primary studies, systematic reviews and meta‐analyses pertinent to our research topic. The search strategy employed a combination of Medical Subject Headings (MeSH) and keywords. These included ‘tamsulosin’, ‘alpha blockers’, ‘mirabegron’, ‘adrenergic beta‐3 agonist’, ‘stent’, ‘discomfort’, ‘pain’, ‘complication’, ‘ureter’, ‘ureteral’ and ‘randomized controlled trial’. This approach was designed to ensure a comprehensive retrieval of relevant literature, encompassing all pertinent studies related to the management of SRSs using tamsulosin and mirabegron.

### 2.3 Selection of studies, data extraction and assessment of risk of bias

**FIGURE 1:**
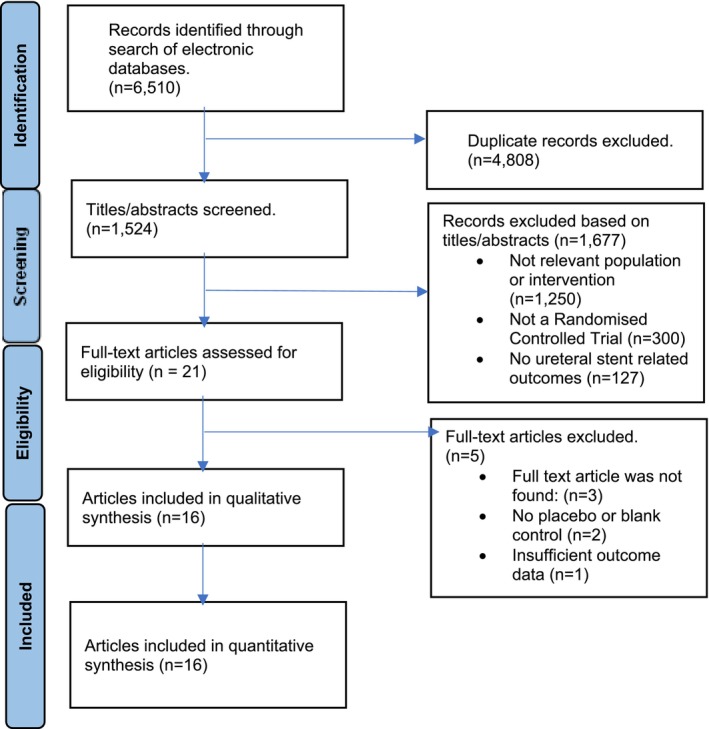
Flow chart of study identification and inclusion process.

The study selection and data extraction processes were meticulously conducted by two independent reviewers (D.M. and A.H., Figure 1). Utilizing the comprehensive search strategy outlined earlier, titles and abstracts of potential literature were identified. Articles deemed suitable during this initial screening underwent full‐text review to determine their compliance with our predefined eligibility criteria. To systematically capture study details, an electronic data extraction spreadsheet was developed. Data extracted from each study included the first author's name, publication year, country of origin, journal, study design, stent indication, intervention, sample size, gender demographics, mean age, analgesic use, stent characteristics and outcome measures (USSQ, IPPS, QOL, analgesia use and adverse events), along with follow‐up duration. In cases of disagreement, a third author served as an adjudicator to ensure impartial resolution.

For assessing the risk of bias in the included randomized trials, we employed the Cochrane Collaboration's tool,^6^ as applied by two independent authors. This tool critically evaluates the quality of studies across multiple domains, including risks associated with the randomization process, deviations from intended interventions, missing outcome data, measurement of outcomes and the selection of reported results. In instances of divergent opinions regarding bias assessment between the two authors, an independent third author intervened to provide an unbiased resolution.

### 2.4 Summary measures, outcome synthesis and sensitivity analyses

We employed Review Manager 5.4.1 (RevMan, Version 5.4.1, Copenhagen, 2020) software to construct a meta‐analysis model for comparing outcomes. A random‐effects model was utilized to determine odds ratio (OR) when assessing dichotomous outcomes and mean difference (MD) when assessing continuous outcomes. To evaluate the heterogeneity among studies for each outcome, we calculated and measured I^2^ using the Cochran's Q test (χ2). Heterogeneity was categorized based on I^2^ percentages: Low heterogeneity was defined as I^2^ ranging from 0% to 25%, moderate heterogeneity from 25% to 75% and high heterogeneity when I^2^ was between 75 and 100%. Additionally, publication bias was assessed visually through the symmetry of funnel plots, specifically for each outcome reported in at least 10 studies. Our comparative meta‐analysis model was underpinned by a 95% confidence interval to establish statistical significance.

To investigate potential sources of heterogeneity and evaluate the robustness of our results, sensitivity analyses were conducted. For each outcome parameter, we repeated the primary analysis using both random‐effects and fixed‐effect models to ensure comprehensiveness. Furthermore, to assess the individual impact of each study on the overall effect size and heterogeneity, we conducted a series of iterative analyses, excluding one study at a time.

## 3 RESULTS

The initial search across electronic databases yielded a total of 1524 articles. After a preliminary screening, 1503 of these were excluded for being irrelevant to our study's topic. Subsequent in‐depth examination of the full texts of the remaining 21 articles led to the exclusion of three, due to the unavailability of the full text, and an additional two were excluded for lacking a placebo/control group. Ultimately, 16^7^, ^8^, ^9^, ^10^, ^11^, ^12^, ^13^, ^14^, ^15^, ^16^, ^17^, ^18^, ^19^, ^20^, ^21^, ^22^ RCTs met our eligibility criteria (Figure 1), encompassing a combined total of 1635 patients. This cohort included 793 patients in the control group, 541 in the tamsulosin group and 301 in the Mirabegron group, all of whom were suitable for inclusion in our meta‐analysis. Detailed information regarding each study, such as study design, publication date, stent indication in the study populations, intervention therapies and sample sizes, is systematically presented in Table 1. Furthermore, Table 2 reports the baseline demographics and clinical characteristics of the study participants. These include gender distribution, mean age, analgesic use, stent characteristics and outcome measures (USSQ, IPPS, QOL, analgesia use and adverse events), as well as the duration of follow‐up.

**TABLE 1:** Characteristics of included studies.

Study	Country	Journal	Study design	Stent indication	Intervention (mg)	Sample size		
						Total	Trial	Control
Wang 2009	Taiwan	Urologia Internationalis	RCT	URSL	Tamsulosin 0.4	146	75	71
Wang (2) 2009	Taiwan	Urological Research	RCT	URSL	Tamsulosin 0.4	154	79	75
Navanimitkul 2010	Thailand	J Int Med Res	RCT	URSL, PCNL, Retrograde balloon dilatation	Tamsulosin 0.4	42	21	21
Lim 2011	Korea	Korean journal of Urology	RCT	URS	Tamsulosin 0.2	91	43	48
Shelbaia 2011	Egypt	African journal of Urology	RCT	URSL	Tamsulosin 0.4	136	69	67
Shalaby 2013	Egypt	Advances in Urology	RCT	Before ESWL, URS, URSL, PCNL, endoscopic endopyelotomy	Tamsulosin 0.4	163	82	81
Singh 2014	USA	International Urology and Nephrology	RCT	URSL, PCNL	Tamsulosin 0.4	60	30	30
Park 2015	Korea	World Journal of Urology	RCT	URSL	Tamsulosin 0.2	43	20	23
El‐Nahas 2016	Egypt	World Journal of Urology	RCT	URS, URSL	Tamsulosin 0.4	88	44	44
Thakur 2016	Nepal	Journal of society of surgeons of Nepal	RCT	URSL, PCNL	Tamsulosin 0.4	46	23	23
Tae 2018	Korea	BJU International	RCT	URS and RIRS	Mirabegron 50	96	48	48
Tae (2) 2018	Korea	European Urology Supplements	RCT	URS, RIRS	Mirabegron 50	58	26	32
Sahin 2020	Turkey	Archivos espanoles de urologia	RCT	URS and external compression	Mirabegron 50	80	40	40
Galal 2021	Egypt	Central European Journal of Urology	RCT	URS/RIRS	Mirabegron 50	206	103	103
Yavuz 2021	Turkey	Lower Urinary Tract Symptoms	RCT	URSL	Tamsulosin 0.4 Mirabegron 50	161	55 50	56
Abdelaziz 2022	Egypt	World Journal of Urology	RCT	URS, RIRS	Mirabegron 50	65	34	31

Abbreviations: ESWL, extracorporal shock wave lithotripsy; PCNL, percutaneous nephrolithotomy; RCTs, randomized controlled trials; RIRS, retrograde intrarenal surgery; URS, ureteroscopy; URSL, ureteroscopic lithotripsy.

**TABLE 2:** Patient demographics, clinical characteristics, and trial outcomes.

Study	Gender	Mean age	Analgesic	Stent			Outcomes					Follow‐up duration (weeks)
	Male/female			Type	Diameter (Fr)	Size (cm)	Analgesia use	USSQ	IPPS total	Quality of life	Adverse event	
Wang 2009	‐C: 55/16 ‐T: 61/14	C: 50.8 T: 50.4	Buprenorphine on demand	Double‐J silicone coated ureteral sent	7	26(Fixed)	‐C:0.07 ± 0.13 ‐T:0.01 ± 0.05	NR	NR	C 4.21 + −0.89. T1.6 + −0.7	C:11 T: 4	2
Wang (2) 2009	‐C: 59/16 ‐T: 63/16	C: 51.5 ± 11 T: 50.1 ± 9.7	Buprenorphine on demand	Double‐J silicone coated ureteral sent	7	Adjusted	‐C:0.096 ± 0.16 ‐T:0.01 ± 0.04	‐Urinary symptoms: C:31.59 + −4.69 T:20.96 + −3.38 ‐Body pain: C:13.3 ± 13.3 T:9.94 ± 9.65 ‐general health: C:12.2 ± 2.99 T:10.1 ± 2.31 ‐ Work performance: C:5.57 ± 1.91 T11 ± (6–17) ‐ Sexual matters: C4.23 ± 1.56. T3.65 ± 1.20	NR	C4.21 + −0.89. T1.6 + −0.74	C:9 T:3	1
Navanimitkul 2010	‐C: 6/15 ‐T: 9/12	C: 51.5 T: 46.1	Paracetamol on demand	Biocompatible polyurethane with a HydroPlusTM coating	6	Adjusted	NR	NR	C:12.67 ± 1.12. T:6.86 ± 0.95	C3.38(0.305) T1.71(0.31)	C:0 T:2	2
Lim 2011	‐C: 31/17 ‐T:24/19	C: 50.08 ± 11.47 T: 49.91 ± 15.23	NR	Polyurethane	6	Adjusted	NR	NR	C:13.77 ± 4.5. T:12.77 ± 5.24	NR	NR	2
Shelbaia 2011	‐C: 44/24 ‐:50/17	C: 29 T: 35	Paracetamol on demand	NR	6	Fixed (26)	NR	NR	T:16.5 ± 3. C: 38.7 ± 4.65	C5.1 + −0.8. T8.8 + −1	C:0 T:5	4
Shalaby 2013	NR	C: 44.0 ± 15.2 T: 41.3 ± 17.1	NR	Polyurethane	Variable	Adjusted	NR	NR	C:15.46 ± 4.28 T:12.40 ± 4.50	C: 4.12 ± 1.76 T: 2.80 ± 1.52	NR	**2**
Singh 2014	*NR*	C: 31.43 ± 9.45 T: 32.70 ± 11.71	Diclofenac and/tramadol on demand	Polyurethane	Variable	Adjusted	NR	‐Urinary symptoms: C:21.7 ± 4.78. T:16.43 ± 5.8 ‐Body pain: C:16.66 ± 4.27. T:13.16 ± 3.24. ‐General health: C:13.5 ± 2.82. T:12.46 ± 2.67. ‐Work performance: C:8 ± 1.28. T:6.86 ± 1.38 ‐ Sexual matters: C:0.86 ± 1.65. T:0.43 ± 1.33	NR	C2.16 + −0.83. T1.76 + −0.56	C:0 T:6	4
Park 2015	‐C: 14/9 ‐T: 9/11	C: 48.7 + −9.0 T: 54.5 + −13.4	Paracetamol and Tramadol on demand	Percuflex, Boston scientific	Fixed (6)	Adjusted	C: 8.8 ± 4.3 T:7.1 ± 5.1	‐Urinary symptoms: C:29.3 ± 7.5. T:31.8 ± 8.1 ‐Body pain: C:19.6 ± 9.4 T:18.7 ± 10.3 ‐General health: C:12.2 ± 4.4 T:15 ± 6.3 ‐Work performance: C:5.5 ± 2.1 T:7.8 ± 3.5 ‐ Sexual matters: C:3.2 ± 1 T:3 ± 1	NR	C: 4.2 ± 1.5 T: 5.2 ± 1.8	C:0 T:2	**2**
El‐Nahas 2016	C: 24/16 T: 19/21	C: 40.8 ± 9 T: 41.4 ± 7.9	NR	Percuflex, Boston scientific	Fixed (6)	Adjusted	NR	‐Urinary symptoms: C:31.7 ± 5.4 T:29.6 ± 3.7 ‐Body pain: C:19.4 ± 4.8 T:17.6 ± 3.5 ‐General health: C:17 ± 3.4 T:16.6 ± 3 ‐Work performance: C:9.5 ± 2.5 T:9.3 ± 2.4 ‐ Sexual matters: C:5.1 ± 3.7 T:2.4 ± 2.7	NR	NR	NR	1–2
Thakur 2016	C: 12/11 T: 10/13	C: 36.43 ± 10.99 T: 37.96 ± 12.98	Paracetamol on demand	NR	NR	NR	C: 27.87 ± 7.66 T: 13.78 ± 4.91	NR	C:14.74 ± 5.25. T: 4.70 ± 3.89	C:5.43 ± 0.59 T: 2.87 ± 1.14	NR	2
Tae 2018	C: 22/26 M: 20/28	C: 50.21 ± 11.74 M: 52.92 ± 12.92	Ibuprofen on demand	Percuflex, Boston scientific	Fixed (6)	Adjusted	‐C:5.5 ± 1.37 ‐M:2.6 ± 2.37	‐Urinary symptoms: C:32.58 ± 6.67 M:27.92 ± 7.72 ‐Body pain: C: 21.96 ± 9.81. M: 13.96 ± 7.24. ‐General health: C:17.71 ± 5.52 M:14 ± 5.16 ‐ Work performance: C:7.25 ± 4.79. M:6.17 ± 3.77 ‐ Sexual matters: C:1.69 ± 2.79. M:1.45 ± 2.33	C:18.04 ± 8.35. M:14.37 ± 8.47	C 4.08 + −1.27, M3.58 + −1.40	C:3 M:3	2
Tae (2) 2018	C: 18/14 M: 10/16	C: 48.31 ± 9.97 M:52.23 ± 14.96	NR	NR	NR	NR	C:2.0 ± 2.66 M:0.38 ± 1.36	‐Urinary symptoms: C:33.19 ± 5.95 M:24.38 ± 6.54 ‐Body pain: C:11.50 ± 9.52 M: 2.62 ± 3.87 ‐General health: C:16.94 ± 6.45 M:13.38 ± 5.69	C: 18.44 ± 8.14 M:10.08 ± 6.86	C:4.25 ± 1.55 M:3.23 ± 1.07	NR	2 ± 2
Sahin 2020	C: 22/18 M: 20/20	C:41.74 ± 12.59 M: 41.7 ± 12.24	NR	NR	Fixed	Fixed	NR	NR	C 21.78 + 2.5. M13.65 + −4.97	NR	NR	6
Galal 2021	‐C: 78/25 ‐M:68/35	C: 46.6 ± 8.2 M: 44.7 ± 9.4	On demand details NR	Polyurethane	Fixed	Adjusted	‐C:0.74 ± 1.29 ‐M:0.30 ± 0.79	NR	NR	C 3.27 + −1.32. M2.79 + −1.05	C:2 M:3	2
Abdelaziz 2022	C: 19/12 M: 23/11	C: 38.1 ± 11.6 M:37.3 ± 10.3	Diclofenac on demand	Polyurethane	Fixed	Adjusted	NR	‐ Urinary symptoms: C: 31 ± 4 M:13 ± 6 ‐Body pain: C: 17.9 ± 6 M:12.5 ± 6 ‐General health: C: 16.4 ± 5 M: 13.7 ± 5 ‐ Work performance: C: 6.3 ± 3.6 M: 5.7 ± 3.6 ‐ Sexual matters: C: 2.9 ± 2 M: 2.1 ± 2.2	C: 8.6 ± 3.7 M: 3.6 ± 3.1	C: 4.1 ± 0.5 M: 1.9 ± 0.4	C:2 M:0	2 ± 2

Abbreviations: C, control; M, mirabegron; NR, not reported; T, tamsulosin.

### 3.1 Assessment of risk of bias in included studies

**FIGURE 2:**
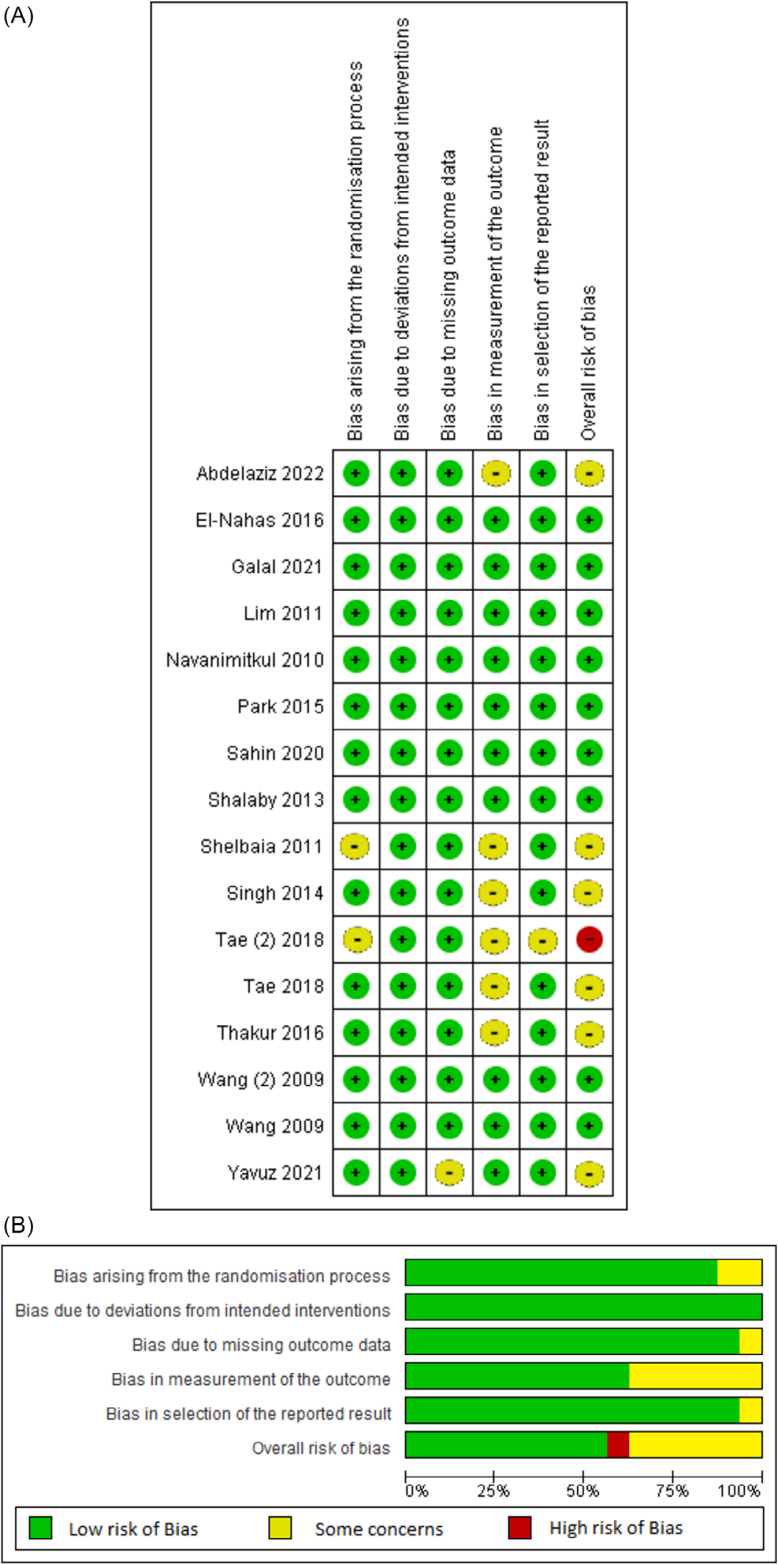
Risk of bias summary and graph showing authors' judgements about each risk of bias item.

The outcomes of risk of bias assessment using Cochrane collaboration's tool are presented in Figure 2.

### 3.2 Outcomes

#### 3.2.1 USSQ

**FIGURE 3:**
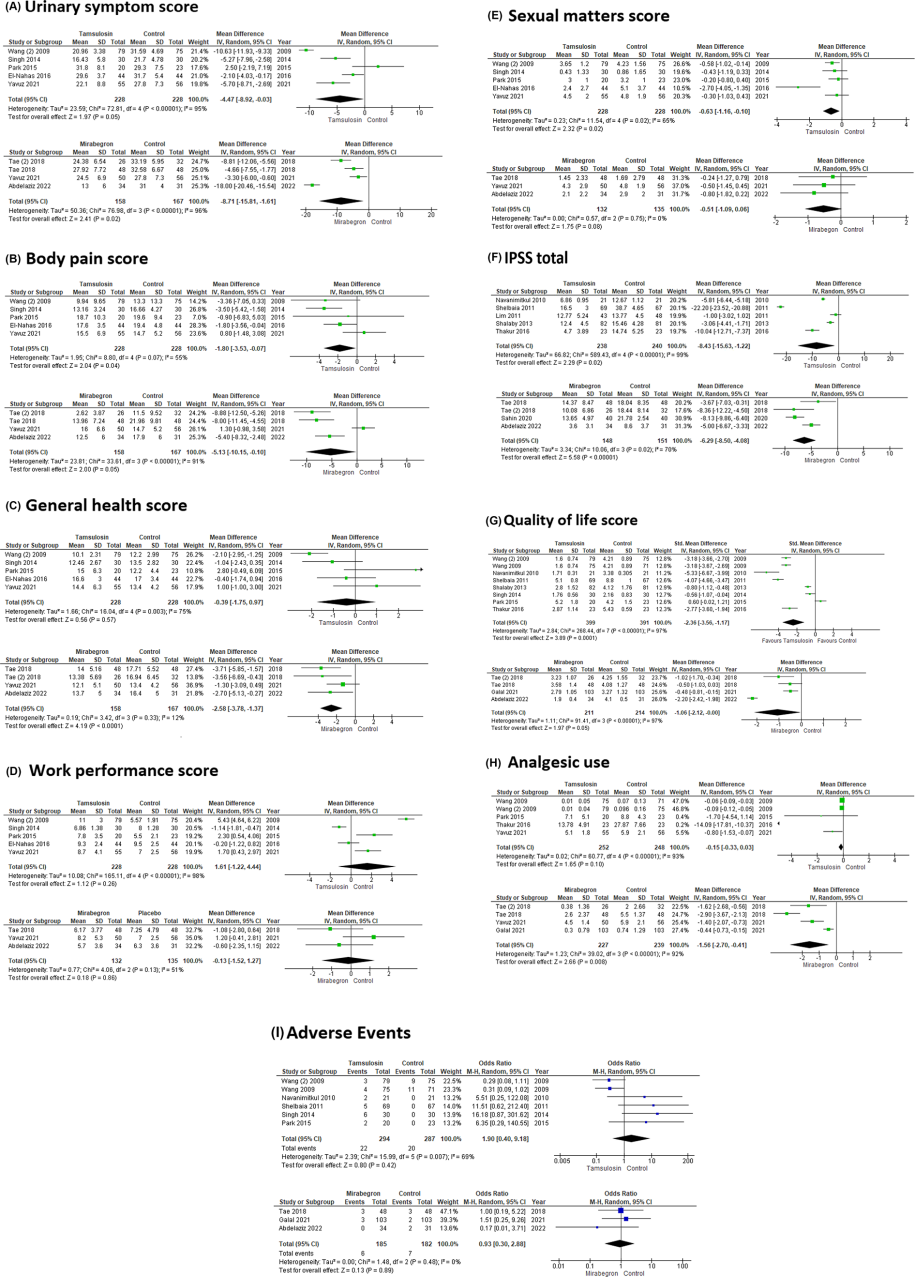
Forest plots of (A) urinary symptom score. (B) Body pain score. (C) General health score. (D) Work performance score. (E) Sexual matters score of Ureteral Stent Symptom Questionnaire. (F) Total International Prostate Symptom Score. (G) Quality of life score. (H) Analgesic use. (I) Adverse events in the tamsulosin/mirabegron and control group.

The USSQ, encompassing six sections (urinary symptoms, body pain, general health, work performance, sexual matters and other problems), is designed to evaluate the extent to which each symptom is bothersome, with higher scores indicating greater severity. Forest plots illustrate changes in these domains (Figure 3A,B,C,D,E).

In our meta‐analysis, we included five studies^8^, ^13^, ^14^, ^15^, ^21^ involving 456 patients (228 in the tamsulosin group and 228 in the control group) to assess the efficacy of tamsulosin using the USSQ. The results indicated that tamsulosin significantly reduced body pain scores (MD −1.80; 95% CI −3.53 to −0.07; *p* = 0.04), and sexual performance scores (MD −0.63; 95% CI −1.16 to −0.10; *p* = 0.02) compared to the control group. However, no significant differences were observed in urinary symptom scores (MD −4.47; 95% CI −8.92 to −0.03; *p* = 0.05), general health (MD 0.39; 95% CI −1.75 to 0.97; *p* = 0.57) and work performance scores (MD 1.61; 95% CI −1.22 to 4.44; *p* = 0.26).

Four studies^17^, ^18^, ^21^, ^22^ involving 325 patients were evaluated to assess the effectiveness of mirabegron (158 in the mirabegron group and 167 in the control group). Mirabegron significantly reduced urinary symptoms (MD −8.71; 95% CI −15.81 to −1.61; *p* = 0.02) and general health scores (MD −2.58; 95% CI −3.78 to −1.37; *p* < 0.0001). No significant difference was noted in body pain (MD −5.13; 95% CI −10.15 to −0.10; *p* = 0.05), work performance scores (MD −0.13; 95% CI −1.52 to 1.27; *p* = 0.86) and sexual performance scores (MD −0.51; 95% CI −1.09 to 0.06; *p* = 0.08).

#### 3.2.2 IPSS

Five studies [9–12, 16] with 478 patients (238 in tamsulosin and 240 in control) and four studies^17^, ^18^, ^19^, ^22^ with 299 patients (148 in mirabegron and 151 in control) were analysed for IPSS. Both tamsulosin (MD −8.4; 95% CI −15.63 to −1.22; *p* = 0.02) and mirabegron (MD −6.29; 95% CI −8.50 to −4.08; *p* < 0.00001) showed significant reductions in total IPSS (Figure 3F). QoL score was reported in eight studies^7^, ^8^, ^9^, ^11^, ^12^, ^13^, ^14^, ^16^ with 790 patients (399 in tamsulosin and 391 in control) and four studies^17^, ^18^, ^20^, ^22^ with 425 patients (211 in mirabegron and 214 in control) were included. Tamsulosin significantly improved QoL scores (MD −2.36; 95% CI −3.56 to −1.17; *p* = 0.0001), while mirabegron demonstrated a trend to improvement, which was not statistically significant (MD −1.06; 95% CI −2.12 to −0.00; *p* = 0.05) Figure 3G.

#### 3.2.3 Analgesic use

Analysis of analgesic use involved five studies^7^, ^8^, ^14^, ^16^, ^21^ with 500 patients (252 in tamsulosin and 248 in control) and four studies^17^, ^18^, ^20^, ^21^ with 466 patients (227 in mirabegron and 239 in control). A significant decrease in analgesic use was noted in the mirabegron group (MD −1.56; 95% CI −2.70 to −0.41; *p* = 0.008) compared to the control group. Tamsulosin did not show a significant difference in analgesic use (MD −0.15; 95% CI −0.33 to 0.03; *p* = 0.10) Figure 3H.

#### 3.2.4 Adverse events

Six studies^7^, ^8^, ^9^, ^11^, ^13^, ^14^ with 581 patients (294 in tamsulosin and 284 in control) and three studies^17^, ^20^, ^22^ with 367 patients (185 in mirabegron and 182 in control) were assessed for adverse events. The analysis revealed that neither tamsulosin (OR 1.90; 95% CI 0.40–9.18; *p* = 0.42) nor mirabegron (OR 0.93; 95% CI 0.30–2.88; *p* = 0.89) was associated with a significant increase in adverse events Figure 3I.

#### 3.2.5 Sensitivity analysis

Switching from a random effect to a fixed‐effect model did not affect the pooled effect size in any of the outcomes. Specific study removals yielded notable shifts in effect sizes:

- The omission of Tae et al.^18^ altered the direction of the urinary symptoms score towards non‐significance for mirabegron.
- The removal of studies by El‐Nahas et al.,^15^ Park et al.,^14^ Singh et al.,^13^ and Wang et al.^8^ made the body pain score's effect size shift towards non‐significance for tamsulosin.
- The withdrawal of Wang et al.'s^8^ study moved the sexual performance score's effect size towards non‐significance for tamsulosin.
- The removal of studies by Thakur et al.^16^ and Navanimitkul et al.^9^ altered the total IPSS effect size direction towards non‐significance for tamsulosin.
- The exclusion of Yavuz et al.'s [21]study altered the analgesic use effect size towards non‐significance for mirabegron.

## 4 DISCUSSION

The indispensable role of ureteral stents in endourological procedures is well recognized, primarily for their utility in ensuring unobstructed urinary flow postoperatively. However, the ubiquity of SRSs cannot be overlooked, with 80% of patients reporting lower urinary tract symptoms (LUTS) and pain, 58% reporting diminished work capacity and 32% reporting sexual dysfunction, underscoring the clinical challenge of managing these adverse effects.^1^, ^21^ The aetiology of SRSs is multifaceted, with current understanding pointing to ureteral and trigonal irritation, stent‐induced urinary reflux and ureteric spasms as contributing factors.^23^

Pharmacological interventions, notably analgesics, anticholinergics and alpha‐blockers, have emerged as the cornerstone for SRS management.^2^, ^3^, ^24^, ^25^ Among these, the alpha‐blocker tamsulosin has been rigorously evaluated and shown to ameliorate SRSs effectively.

Chen et al.^2^, ^3^ conducted a meta‐analysis evaluating the effectiveness of tamsulosin monotherapy in reducing SRSs. Pooled results showed that tamsulosin could significantly reduce urinary symptoms and pain and improve sexual performance compared to placebo.^3^ Similarly, Li et al.'s investigation into mirabegron's utility in SRS management highlighted its superiority over control treatments in improving urinary symptoms and general health indices and reducing analgesic dependency.^23^ Both studies concluded that tamsulosin and mirabegron monotherapy were effective in relieving SRSs.

To our knowledge, this is the first meta‐analysis to juxtapose the efficacy and safety profiles of mirabegron and tamsulosin in a comparative analysis across standardized domains including, USSQ, IPPS, QoL analgesia use and adverse events. Tamsulosin and mirabegron have been proven to alleviate SRSs; however, it is still unclear which drug is more effective in which domain.

Our findings demonstrate that tamsulosin shows a statistical significance in alleviating body pain, enhancing sexual function, and QoL, whereas mirabegron shows statistical significance in ameliorating urinary symptoms and general health and reducing analgesia use. Noteworthy is the observation that both agents showed a trend towards improvement in certain domains, albeit not reaching statistical significance. Specifically, tamsulosin showed a near‐significant effect on urinary symptoms (*p* = 0.05), while mirabegron indicated potential benefits for body pain, sexual performance and QoL, with *p*‐values of 0.05, 0.08, and 0.05, respectively. Moreover, both medications were associated with statistically significant improvements in the IPPS, without notable impact on work performance.

In terms of safety, our comparative analysis revealed no significant disparity in the incidence of adverse events between tamsulosin or mirabegron and their respective control groups. The adverse events associated with tamsulosin, as identified in the included studies, encompassed transient hypotension, dizziness, headache, asthenia, syncope, retrograde ejaculation and palpitations. Conversely, mirabegron was linked to constipation, dry mouth and diarrhoea. It is important to note that such adverse events were infrequent and generally mild in nature. Furthermore, none of the trials within our analysis reported severe adverse events necessitating participant withdrawal. This observation supports the conclusion that mirabegron is comparable to tamsulosin in terms of both efficacy and safety for managing SRSs.

Several recent studies have explored the direct comparison between these two medications. For instance, Alexander et al.^26^ and Chandna et al.^27^ found that while both drugs are effective, tamsulosin outperforms mirabegron in certain domains, particularly urinary symptom relief, whereas mirabegron shows advantages in other areas such as sexual function and general health. Similarly, Javid et al.^28^ reported that mirabegron was more effective in reducing urinary symptoms and body pain compared to tamsulosin. These findings support the complementary roles that these medications may play in managing diverse symptoms associated with ureteral stents. In addition to these direct comparisons, Xiang et al.^29^ conducted a **network meta‐analysis** comparing tamsulosin, mirabegron and solifenacin for managing ureteral SRSs. This analysis confirmed that all three medications were significantly better than placebo in improving urinary symptoms. Interestingly, **solifenacin** ranked highest for **urinary symptom relief,** followed closely by **mirabegron**, while **tamsulosin** ranked lower in this domain. However, **mirabegron** was superior in alleviating **body pain**, improving **sexual matters** and minimizing adverse events. While network meta‐analyses provide useful insights through indirect comparisons, our meta‐analysis, which directly compares each drug to placebo, offers a clearer understanding of their absolute effectiveness in clinical practice, aiding clinicians in deciding when to initiate treatment. Furthermore, while this meta‐analysis focused on individual drug efficacy, the potential for **combination therapy** has been explored. Zhang et al.^30^ examined the combined use of tamsulosin and mirabegron but found no significant additional benefit compared to tamsulosin monotherapy. Despite this, the complementary mechanisms of these two drugs suggest that combination therapy may still be beneficial in specific patient subgroups, especially those with more complex or severe symptom profiles. Future research should explore the potential advantages of combination therapy in these populations.

Our sensitivity analysis revealed discrepancies. The exclusion of Tae et al.^18^ from our meta‐analysis resulted in a shift from statistical significance to non‐significance for the efficacy of mirabegron to alleviate urinary symptoms. Although this particular study contributed the least weight due to its relatively smaller sample size, its effect size was notably large and favoured mirabegron significantly, which had a disproportionate influence on the pooled results. Consequently, the removal of Tae et al.,^18^ with its methodological limitations and incomplete reporting, highlights the need for caution in over‐relying on individual studies with significant results that lack comprehensive quality assessment. This underscores the importance of considering both the statistical weight and the quality of evidence when interpreting meta‐analysis outcomes. The exclusion of the studies by El‐Nahas et al.,^15^ Park et al.,^14^ Singh et al.^13^ and Wang et al.^8^ from our meta‐analysis has led to a shift towards non‐significance for the effect of tamsulosin on body pain scores. These studies, despite varying in analgesic use, follow‐up duration and reporting quality, contribute significantly negative mean differences that are consistent with a beneficial effect of tamsulosin. Park et al.,^14^ while having a confidence interval that includes zero, still contribute a negative effect size, and its removal, along with the others, diminishes the pooled mean difference. Moreover, the Yavuz et al.^21^ study, with a longer follow‐up period and different analgesia, reported a positive mean difference, which diverges from the combined negative effect of the other studies. These discrepancies in methodology and outcomes may account for Yavuz et al.^21^ deviation from the trend and its influence on the meta‐analysis results, underscoring the importance of methodological homogeneity in interpreting pooled data. Removing the studies by Wang et al.,^8^ Thakur et al.^16^ and Navanimitkul et al.^9^ from the meta‐analysis notably changed the significance of tamsulosin's effects on sexual performance and IPSS scores. These studies' large effect sizes and weights were key to the initial findings; their exclusion diminished the evidence and broadened confidence intervals, resulting in non‐significant pooled estimates. Furthermore, SRS may be more prevalent in procedures performed from below, such as URS, than from above, like PCNL. These studies incorporated PCNL procedures and reported more favourable IPSS scores, Illustrating how type of procedure can affect SRS outcome. Consequently their exclusion significantly alters the pooled significance, pointing to an undervalued benefit of tamsulosin. The removal of the Yavuz et al.^21^ study from the meta‐analysis significantly affected the overall effect size regarding analgesic use for mirabegron. Given its longer follow‐up period of 4 weeks, the Yavuz et al.^21^ study may better capture the sustained effects of mirabegron on reducing the need for analgesics as patients recover from stent placement. Its exclusion eliminates a unique perspective on the longer term use of analgesics, which contrasts with the shorter 2‐week duration of the other studies. This could lead to an underestimation of mirabegron's benefits over a more extended period, thus shifting the pooled effect size towards non‐significance when Yavuz et al.^21^ are not considered.

The sensitivity analysis conducted in our meta‐analysis revealed potential implications of the small sample sizes and limited number of studies included in our review. While our study provides valuable insights into the comparative effectiveness of tamsulosin and mirabegron for managing ureteral SRSs, the modest sample sizes and the relatively small number of studies may have influenced the precision and generalisability of our findings. Given the promising results observed in our meta‐analysis, there is a clear need for further research in this space to elucidate the full potential of combining tamsulosin and mirabegron in treating SRSs. We advocate for the initiation of high‐calibre, prospective, double‐blinded RCTs to explore the synergistic effects of these medications in treating SRSs, aiming to optimize patient outcomes and QOL. Despite these methodological challenges, this meta‐analysis serves as a pivotal contribution to the evolving landscape of SRS management, offering an initial comparative insight into the efficacy and safety profiles of mirabegron and tamsulosin.

## 5 CONCLUSION

This meta‐analysis substantiates the efficacy and safety of both tamsulosin and mirabegron in the management of SRSs, characterized by a minimal incidence of adverse events. Mirabegron notably excels in alleviating urinary symptoms, enhancing general health and reducing the reliance on analgesics, whereas tamsulosin demonstrates pronounced benefits in mitigating body pain, improving sexual function and elevating the QoL. Specifically, mirabegron demonstrated significant improvements in areas where tamsulosin did not show notable efficacy, and vice versa. The distinct efficacy profiles of these medications in specific clinical domains hint at a potential complementary therapeutic approach, with each agent offering unique benefits. Given the promising findings, we advocate for the initiation of high‐calibre, prospective, double‐blinded RCTs to explore the synergistic potential of combining tamsulosin and mirabegron in treating SRSs, aiming to optimize patient outcomes and QOL.

## AUTHOR CONTRIBUTIONS

D.M. and K.P. conceived of and designed the methodology. A.H. and N.C.S. contributed to the literature search, study selection, data extraction, and risk of bias assessment. D.M. conducted the meta‐analysis and drafted the manuscript with critical review by K.P. and K.S. All authors reviewed and approved the final manuscript.

## CONFLICTS OF INTERESTS STATEMENT

The authors declare no conflict of interest.

## Supporting information


**Table S1.** Search Strategy Table.
