# Supplementary material for: Ureteral stent symptoms: A systematic review and meta‐analysis comparing the use of mirabegron and tamsulosin
Source: BJUI Compass. 2025 Sep 11;6(9):e2485. doi: 10.1002/bco2.485 (PMC12425636; doi:10.1002/bco2.485)
Supplement: Supplementary file 1 — Table S1. Search Strategy Table. [file BCO2-6-e2485-s001.docx]

**
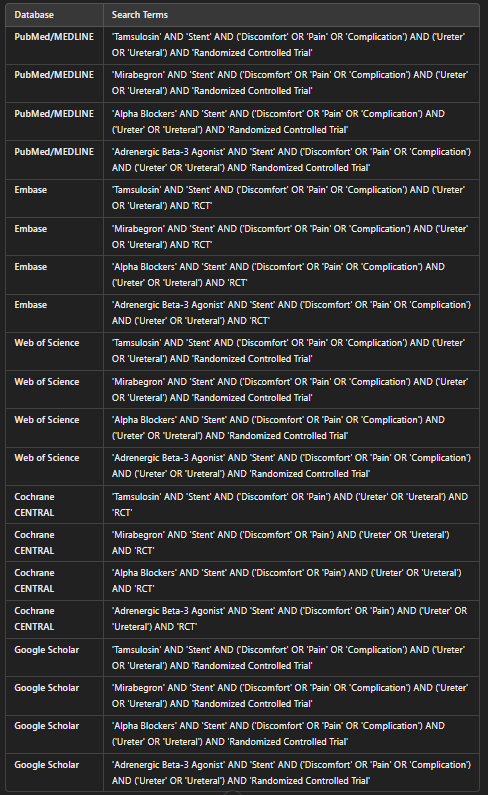
Search Strategy Table:**

This search strategy was adopted for following databases: PUBMED/MEDLINE, EMBASE, WEB OF SCIENCE, Cochrane Central Register of Controlled Trials (CENTRAL) and GOOGLE SCHOLAR
